# Structural basis for the interaction of SARS‐CoV‐2 virulence factor nsp1 with DNA polymerase α–primase

**DOI:** 10.1002/pro.4220

**Published:** 2021-11-12

**Authors:** Mairi L. Kilkenny, Charlotte E. Veale, Amir Guppy, Steven W. Hardwick, Dimitri Y. Chirgadze, Neil J. Rzechorzek, Joseph D. Maman, Luca Pellegrini

**Affiliations:** ^1^ Department of Biochemistry University of Cambridge Cambridge CB2 1GA UK; ^2^ Present address: The Francis Crick Institute London NW1 1AT UK

**Keywords:** cryo‐EM, DNA polymerase α–primase, nsp1, protein–protein interaction, SARS‐CoV‐2

## Abstract

The molecular mechanisms that drive the infection by the severe acute respiratory syndrome coronavirus 2 (SARS‐CoV‐2)—the causative agent of coronavirus disease 2019 (COVID‐19)—are under intense current scrutiny to understand how the virus operates and to uncover ways in which the disease can be prevented or alleviated. Recent proteomic screens of the interactions between viral and host proteins have identified the human proteins targeted by SARS‐CoV‐2. The DNA polymerase α (Pol α)–primase complex or primosome—responsible for initiating DNA synthesis during genomic duplication—was identified as a target of nonstructural protein 1 (nsp1), a major virulence factor in the SARS‐CoV‐2 infection. Here, we validate the published reports of the interaction of nsp1 with the primosome by demonstrating direct binding with purified recombinant components and providing a biochemical characterization of their interaction. Furthermore, we provide a structural basis for the interaction by elucidating the cryo‐electron microscopy structure of nsp1 bound to the primosome. Our findings provide biochemical evidence for the reported targeting of Pol α by the virulence factor nsp1 and suggest that SARS‐CoV‐2 interferes with Pol α's putative role in the immune response during the viral infection.

## Abstract 2

PDB Code(s): 7OPL;

EMDB codes: 13020, 13021

## 1 INTRODUCTION

The enormous disruption to public health and the global economy caused by COVID‐19 has spurred international research efforts to unravel the molecular biology of SARS‐CoV‐2 in order to devise effective means of therapeutic and prophylactic intervention.

Coronaviruses comprise a large family of positive‐sense single‐strand RNA viruses that cause respiratory and enteric diseases in animals and humans.^1^, ^2^, ^3^ Seven coronaviruses that are capable of infecting humans have been identified to date, belonging to the genera *alphacoronavirus* (HCoV‐229E and HCoV‐NL63) and *betacoronavirus* (HCoV‐OC43, HCoV‐HKU1, MERS, SARS‐CoV, and SARS‐CoV‐2).^4^, ^5^ Within the *betacoronavirus* genus, HCoV‐OC43 and HCoV‐HKU1 generally cause only a mild illness; in contrast, infection with MERS‐CoV, SARS‐CoV, and SARS‐CoV‐2 results in a severe respiratory disease in some patients, with significantly increased fatality rates.

SARS‐CoV‐2 is most closely related to SARS‐like coronaviruses isolated from horseshoe bats found in Southeast Asia; it shares ~79% of its sequence with SARS‐CoV and ~ 50% with MERS‐CoV. Since its initial identification and characterization,^6^, ^7^ genomic sequencing has shown that the SARS‐CoV‐2 genome encodes—from its 5′ to the 3′ end—two large polyproteins 1a and 1ab that are cleaved by viral proteases to generate 16 nonstructural proteins (nsp1–nsp16), four structural proteins (S, E, M, and N), and a number of other accessory proteins.

Upon viral infection, the host cell mounts a rapid response of its innate immune system via the interferon (IFN) pathway, resulting in the secretion of type‐I and type‐III IFNs and subsequent activation of a signaling pathway that leads to the expression of a wide range of IFN‐stimulated genes.^8^ A hallmark of COVID‐19 is the attenuation of the IFN response of the host coupled to high levels of inflammatory cytokine production.^9^ SARS‐CoV‐2 deploys multiple mechanisms to suppress the innate immune response, with participation of several viral proteins including nsp1, nsp6, nsp13, M, ORF3a, ORF6, and ORF7a/b.^10^, ^11^

The nsp1 protein is present in α‐ and β‐coronaviruses but not in γ‐ or δ‐coronaviruses. nsp1 is a major virulence factor for SARS and MERS, due its pleiotropic activities as potent antagonist of virus‐ and IFN‐dependent signaling and suppressor of host mRNA translation.^12^, ^13^, ^14^, ^15^ While there is poor sequence conservation between the nsp1 proteins from α‐ and β‐coronaviruses, both have been shown to induce strong suppression of host gene expression, albeit possibly by different mechanisms.^16^, ^17^, ^18^, ^19^ Despite poor sequence identity, α‐CoV nsp1 and β‐CoV nsp1 share structural homology in their core domain, which folds in a 6‐stranded β‐barrel structure with one α‐helix on the rim of the barrel.^20^, ^21^, ^22^, ^23^, ^24^ The importance and role of nsp1's globular domain are not yet fully understood, although it has recently been shown to interact with the 5 ′‐UTR of the SARS‐CoV‐2 mRNA, helping it escape translational inhibition in infected cells.^25^ In addition to their globular domain, SARS‐CoV and SARS‐CoV‐2 nsp1 proteins contain a long, unstructured C‐terminal extension. A two‐helix hairpin motif near the end of the C‐terminal tail of SARS‐CoV‐2 nsp1 has been shown to bind the 40S subunit of the ribosome and block the mRNA entry channel, providing a structural basis for the role of nsp1 in suppressing host translation in infected cells.^16^, ^26^, ^27^, ^28^

Recently published studies of the human proteins targeted by SARS‐CoV‐2 reported a physical association between nsp1 and the primosome,^29^, ^30^ the complex of DNA polymerase α (Pol α) and primase responsible for initiating DNA synthesis in DNA replication.^31^ In addition to its well‐characterized nuclear role in genomic duplication, participation of Pol α in innate immunity has been suggested, based on the observation that disease‐associated mis‐splicing of *POLA1*—coding for the catalytic subunit of Pol α—results in abnormal IFN I response and autoinflammatory manifestations.^32^, ^33^, ^34^ Here, we demonstrate that nsp1 binds directly to the catalytic subunit of Pol α and elucidate their mode of interaction by determining the cryo‐electron microscopy (cryo‐EM) structure of SARS‐CoV nsp1 bound to the human primosome. Our biochemical and structural characterization of the interaction between the virulence factor nsp1 and the primosome suggests that targeting Pol α–primase is part of a novel mechanism driving the SARS‐CoV‐2 infection. Our data further define protein epitopes on nsp1 and Pol α catalytic subunit that could be exploited for the development of small‐molecule inhibitors of their interaction.

## 2 RESULTS

### 2.1 Biochemical analysis of nsp1 binding to the primosome

A proteomic screen of human proteins that interact with SARS‐CoV‐2 proteins first reported the Pol α–primase complex as high‐confidence interactor of SARS‐CoV‐2 nsp1.^29^ A subsequent study expanded this approach to include the SARS‐CoV and MERS proteins and identified the primosome as interactor of the related SARS‐CoV nsp1.^30^ Recently, a further interactome study between viral and host proteins identified the primosome as a high‐confidence target of the SARS‐CoV‐2 nsp1.^35^ As viral–host protein–protein interactions can play essential roles in driving the infection, we sought to confirm the reported interaction using purified, recombinant samples of nsp1 and the primosome.

**FIGURE 1:**
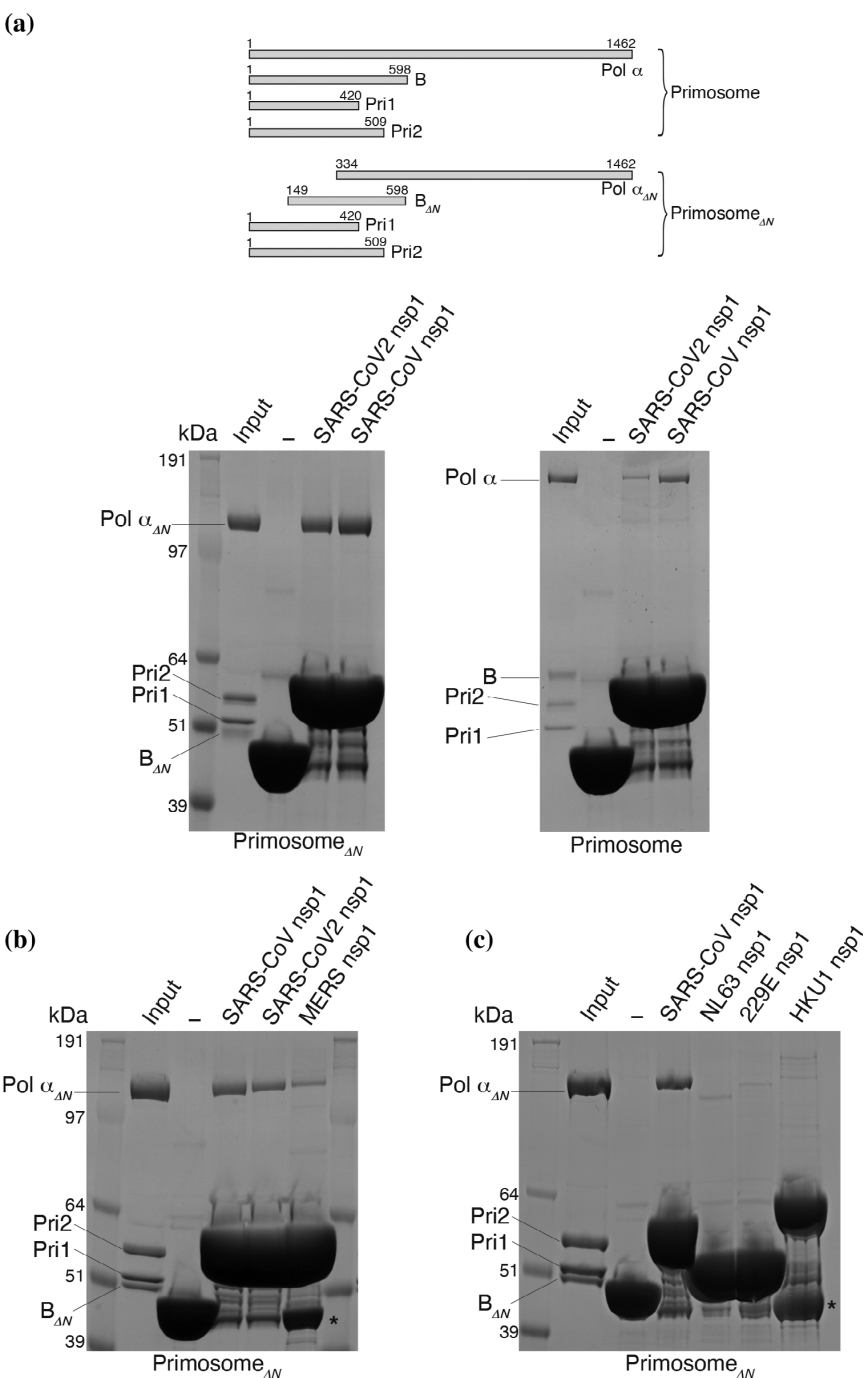
Biochemical reconstitution of the primosome–nsp1 interaction. The panels report the result of pull‐down experiments on amylose beads of human primosome by the indicated nsp1 proteins, N‐terminally fused to maltose‐binding protein (MBP), analyzed by SDS‐PAGE. The lane marked with a dash symbol is the negative control for pull‐down by MBP only. (a) Pull‐down of full‐length and truncated primosomes. A schematic drawing of subunit size for full‐length and truncated primosomes is shown above the panels; (b) pull‐down of truncated primosome with SARS‐CoV, SARS‐CoV‐2, and MERS nsp1; (c) pull‐down of truncated primosome with SARS‐CoV, NLS63, 229E, and HKU1 nsp1. Bands labeled with an asterisk indicate free His_6_‐MBP. nsp1, Nonstructural protein 1; Pol α, DNA polymerase α; SDS‐PAGE, sodium dodecyl sulfate–polyacrylamide gel electrophoresis

Maltose‐binding protein (MBP)‐tagged SARS‐CoV‐2 and SARS‐CoV nsp1 were able to pull down both full‐length and truncated forms of purified primosome (Figure 1a). The truncated primosome lacks the flexible and largely unstructured N‐terminal regions of both Pol α (amino acids 1–333) and B subunit (amino acids 1–148) that are known to mediate several primosome interactions,^36^, ^37^, ^38^, ^39^ thus indicating that the interaction with nsp1 is not dependent on known recognition motifs in Pol α and the B subunit. MERS nsp1 was also able to bind to the primosome, albeit more weakly than the SARS nsp1 proteins (Figure 1b), in accordance with the proteome‐wide interaction studies that identified the primosome as a binding SARS‐CoV and SARS‐CoV‐2 nsp1, but not MERS nsp1.^30^ Importantly, nsp1 from human‐infective coronavirus strains 229E, NL63, and HKU1 did not interact with the primosome in our pull‐down assay (Figure 1c). Thus, the affinity of nsp1 toward Pol α correlates with the ability of the coronavirus strain to cause serious disease.

**FIGURE 2:**
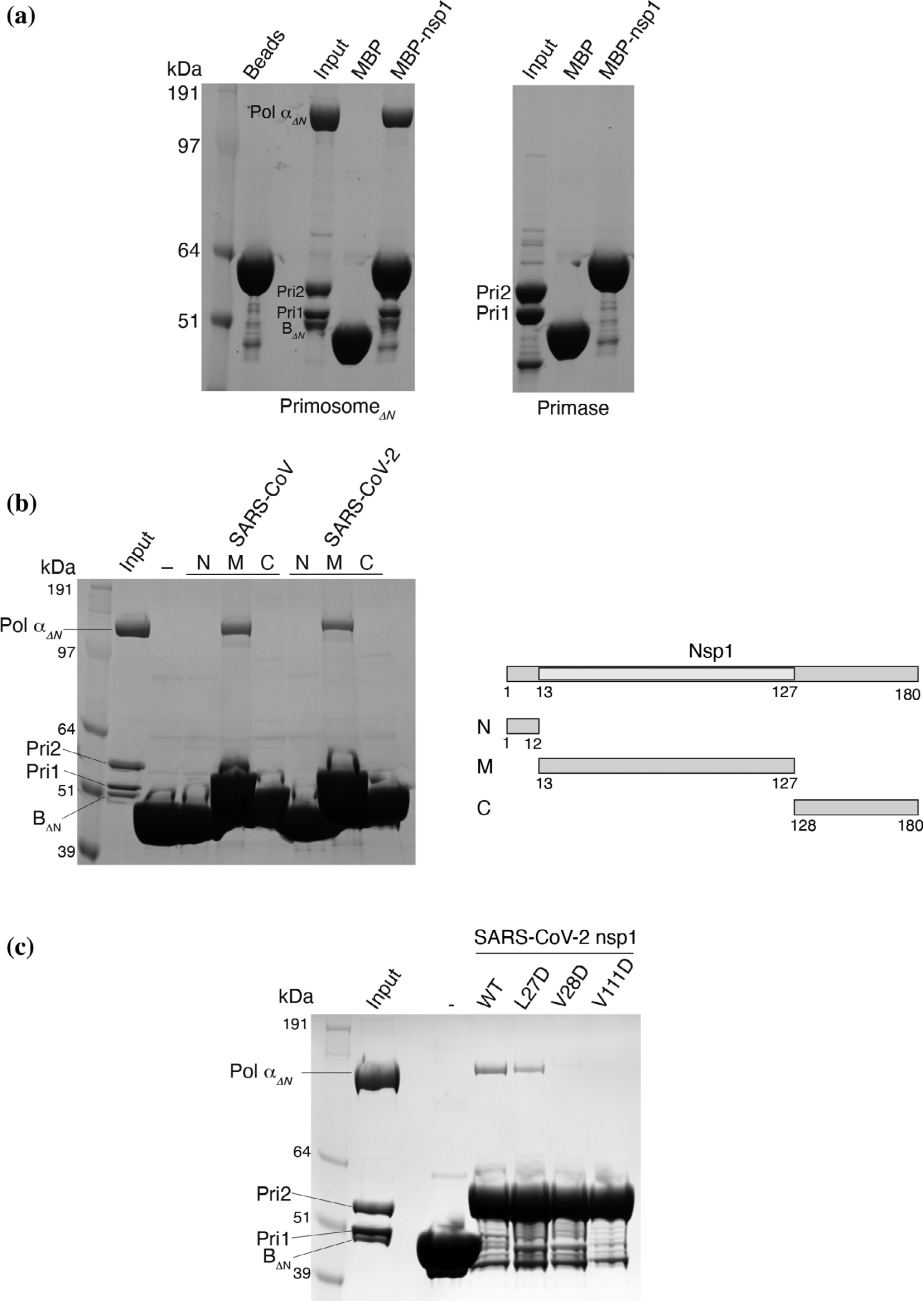
Biochemical dissection of the primosome–nsp1 interaction. The panels report the result of pull‐down experiments on amylose beads of human primosome by the indicated nsp1 proteins, N‐terminally fused to maltose‐binding protein (MBP), analyzed by SDS‐PAGE. The lane marked with a dash symbol is the negative control for pull‐down by MBP only. (a) Pull‐down of truncated primosome and primase; (b) pull‐down of truncated primosome by N‐, M‐, and C‐terminal domains of SARS‐CoV and SARS‐CoV‐2 nsp1 proteins. On the right, schematic diagram of the domain structure of nsp1; (c) pull‐down of truncated primosome by SARS‐CoV‐2 wild‐type and single‐point mutant L27D, V28D, and V111D nsp1 proteins. nsp1, Nonstructural protein 1; Pol α, DNA polymerase α; SDS‐PAGE, sodium dodecyl sulfate–polyacrylamide gel electrophoresis

In the original interaction study,^29^ nsp1 was coprecipitated with all four subunits of Pol α–primase, which are normally found associated as a constitutive complex in the cell. To dissect the interaction between primosome components and SARS‐CoV‐2 nsp1, we performed a pull‐down experiment using MBP‐tagged nsp1 and the primase heterodimer Pri1–Pri2 (Figure 2a). We detected no interaction between nsp1 and primase which, taken together with the data presented in Figure 1a, indicates that the nsp1‐binding site resides within the folded domains of Pol α and the B subunit.

Next, we sought to define the region of nsp1 responsible for the interaction with the primosome. SARS‐CoV and SARS‐CoV‐2 nsp1 fold into a globular middle domain (M; amino acids 13–127) flanked by a short N‐terminal region (N; amino acids 1–12) and a long C‐terminal extension that is predicted to be largely unstructured (C; amino acids 128–180). Pull‐down experiments with the N, M, and C regions of SARS nsp1 showed clearly that the middle domain is responsible for the interaction with the primosome (Figure 2b).

Guided by the published crystal structures of nsp1,^20^, ^23^, ^24^ we identified an exposed hydrophobic surface centered around amino acids L27 and V28 in the first interstrand loop of the β‐barrel in the middle domain and V111 in the last interstrand loop, as a possible primosome‐binding epitope. Point mutations to aspartate—that reversed the chemical nature of the side chain—weakened (L27D) or abolished (V28D and V111D) the interaction of the MBP‐tagged nsp1 mutants with the primosome in our pull‐down assay (Figure 2c), thus implicating these residues in the interaction.

### 2.2 Cryo‐EM structure of the primosome–nsp1 complex

Our findings had shown that a direct biochemical interaction exists between nsp1 and the primosome, confirming the result of the proteomic screens.^29^, ^30^ We therefore decided to elucidate the structural basis for the interaction by determining the high‐resolution structure of the primosome–nsp1 complex by cryo‐EM (Figure S1–S3, Table S1). SARS‐CoV nsp1 was chosen for the structural analysis as this variant showed somewhat stronger binding to human primosome in our pull‐down assays (Figure 1a). We note that there is a high degree of sequence conservation between SARS‐CoV and SARS‐CoV‐2 nsp1 (84.4 and 85.8% identity over the entire sequence and the middle domain only, respectively).

We obtained a 3D reconstruction of the primosome at a global resolution of 3.8 Å after sharpening that clearly showed all components of the complex (Figures S4 and S5). As the portion of the map relative to the primase subunit Pri1 was weaker, a new map was calculated using subtracted particles from which Pri1 had been omitted, yielding a slightly improved map at a global resolution of 3.6 Å after sharpening. Both maps—from full and subtracted particles—were used during model building. Density belonging to the nsp1 middle domain was clearly visible at a lower map threshold than required to visualize the primosome core.

**FIGURE 3:**
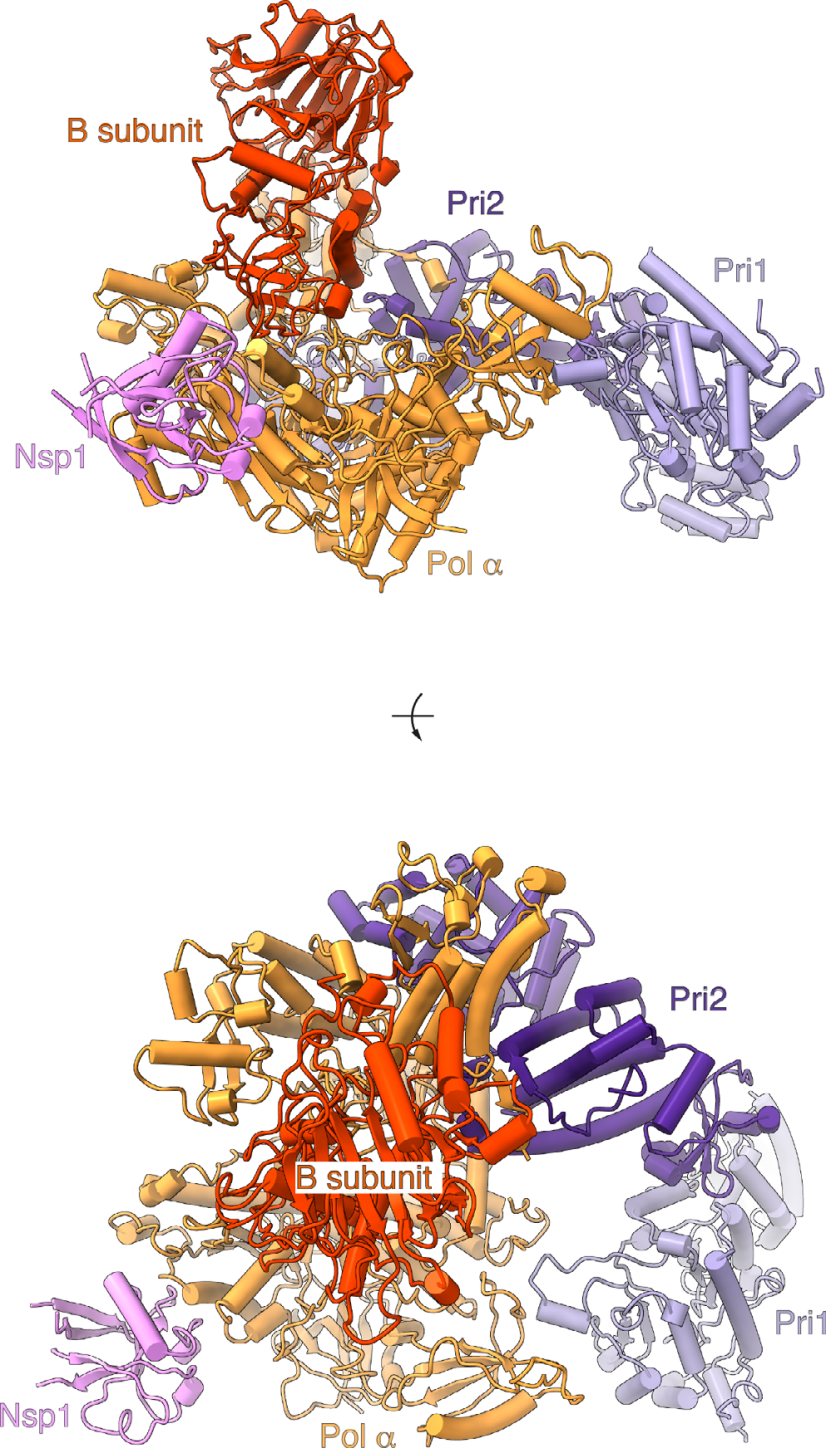
Cryo‐EM structure of the primosome–nsp1 complex. The structure is drawn as a cartoon with helices as solid cylinders and strands as arrows. Each subunit is labeled and uniquely colored, in orange (Pol α), red (B subunit), lilac (Pri1), purple (Pri2), and pink (nsp1). The structure is shown in two views, from the side (top panel) and from the top (bottom panel). Cryo‐EM, cryo‐electron microscopy; nsp1, nonstructural protein 1; Pol α, DNA polymerase α

The cryo‐EM structure of the human primosome confirmed the presence of the binary interfaces that had been previously defined by X‐ray crystallography of yeast and human Pol α–primase subcomplexes.^40^, ^41^, ^42^, ^43^, ^44^ Intriguingly, our cryo‐EM analysis of the primosome revealed a quaternary structure that is incompatible with known and presumed conformations required for activity by its two catalytic subunits, Pol α and primase^42^, ^45^, ^46^, ^47^ (Figure 3). Thus, the B subunit bound to the C‐terminal domain of Pol α is docked within the open “hand” of the polymerase domain, precluding binding of the template DNA–primer RNA duplex. Furthermore, the heterodimeric primase is held in an extended conformation, whereby the Fe‐S domain of Pri2—required for initiation of RNA primer synthesis^48^—is sequestered by interactions with both B and Pol α and unavailable to contact Pri1, which is excluded from the primosome core and largely solvent exposed.

Overall, the cryo‐EM structure is highly similar to a structure of the human primosome determined earlier by X‐ray crystallography^49^ (Figure S6). Although reasonable doubts existed that the crystal structure might have been a crystallization artifact, these have now been dispelled by our cryo‐EM analysis. Altogether, the high‐resolution structural data obtained with X‐ray and cryo‐EM methods indicate that—in its unliganded form—the human primosome exists in a conformation that is incompatible with primer synthesis without the intervention of major conformational rearrangements. Why should the primosome adopt such a distinct quaternary structure in the absence of its DNA and nucleotide substrates and what should be the function of this postulated autoinhibited state^49^ remain unclear.

**FIGURE 4:**
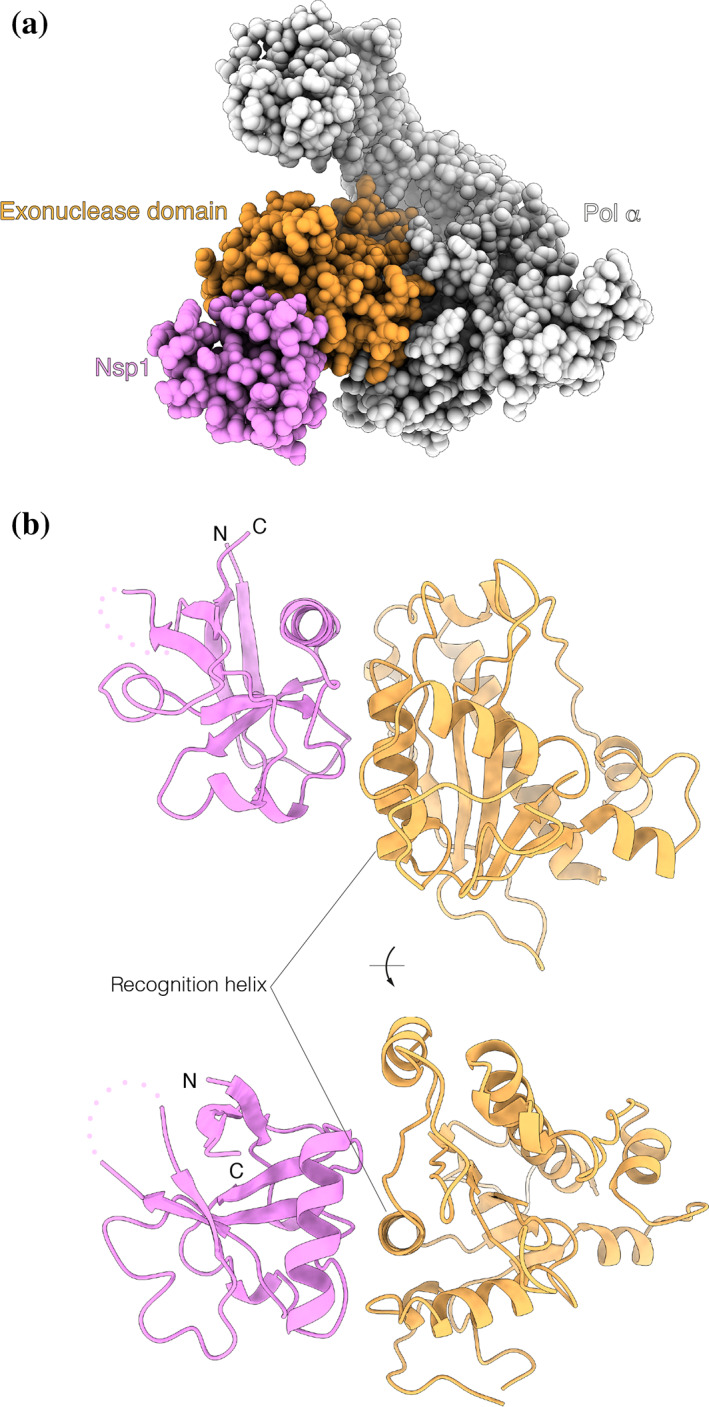
nsp1 binds to the exonuclease domain of Pol α. (a) Space‐fill representation of the interaction between nsp1 (pink) and the inactive exonuclease domain of Pol α (orange) and (b) two views rotated by 90° of the association mode of nsp1 with the exonuclease domain. The proteins are drawn as ribbons in pink (nsp1) and orange (Pol α). A disordered loop of nsp1 facing away from the interface is shown as a dotted line. The position of the recognition helix in the exonuclease domain is indicated. nsp1, Nonstructural protein 1; Pol α, DNA polymerase α

**FIGURE 5:**
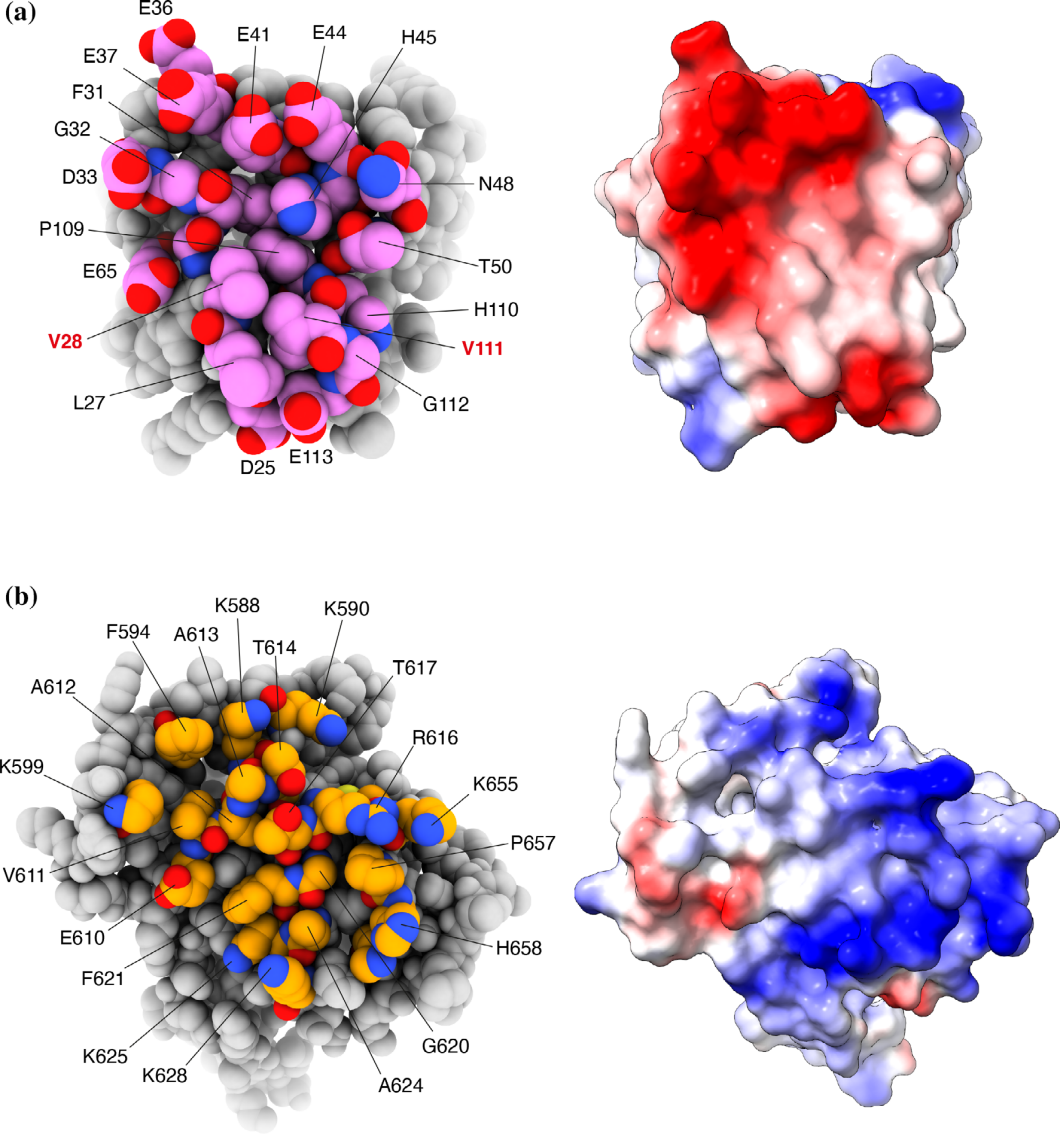
The nsp1–Pol α exonuclease domain interface. (a) On the left, a space‐fill model of nsp1, with interface residues labeled and colored in pink. On the right, the charge distribution of nsp1 mapped over its molecular surface (red: negative charge; blue: positive charge); (b) on the left, a space‐fill model of the exonuclease domain of Pol α, with interface residues labeled and colored in orange, and on the right, the charge distribution of the exonuclease domain mapped over its molecular surface (red: negative charge; blue: positive charge). nsp1, Nonstructural protein 1; Pol α, DNA polymerase α

The structure shows that nsp1 binds to the Pol α subunit of the primosome (Figure 4). The globular domain of nsp1 docks onto the rim of the polymerase fold of Pol α, and its binding site is entirely contained within the inactive exonuclease domain of the polymerase, in agreement with our biochemical findings (Figures 1 and 2). Nsp1 binding is mediated by residues in the first, second, and fourth interstrand loop of the globular β‐barrel domain and in the α‐helix at the C‐end of the second interstrand loop. The interface is of a mixed hydrophobic and polar nature (Figure 5). A cluster of hydrophobic nsp1 residues at the center of the interface—including L27, V28, P109, V111, and G112—becomes buried upon complex formation (Figures 5a and S7), validating the observation that mutations of nsp1 residues V28 and V111 abrogate the interaction (Figure 2c).

The nsp1‐binding area in Pol α is centered on the second α‐helix of the exonuclease domain, comprising residues 615 to 629 (recognition helix), and extends to include solvent‐exposed amino acids flanking the recognition helix, from V585 to I662 (Figures 5b and S7). Hydrophobic amino acids in the exonuclease domain that match the hydrophobic surface of nsp1 in the complex are helical residues A624, G620, and F621, the short aliphatic segment 611‐VAAT‐614 in the loop leading into the recognition helix, and P657 toward the C‐end of the binding interface.

The interaction shows pronounced charge complementarity (Figures 5a,b and S7). A cluster of negatively charged residue in nsp1—D33, E36, E37, E41, and E44 in the helix of the second intrastrand loop, as well as D25, E65, and E113—lines the edge of the interaction area and becomes juxtaposed upon complexation to basic residues K625, K628, and R616 in the recognition helix of the exonuclease domain of Pol α, as well as to surrounding lysine residues K588, K590, K599, K655, and K661.

The presence at the interface of small amino acids such as alanine and glycine, that are required for the intimate association of rather flat protein surfaces, supports the observed interaction between nsp1 and Pol α. Notably, the nsp1‐binding sequence 611‐VAAT‐614, as well as the 620‐GFxxA‐624 motif in the recognition helix, are highly conserved in vertebrate Pol α sequences (Figure S7).

## 3 DISCUSSION

Here, we have provided a biochemical and structural basis for the interaction between the SARS‐CoV‐2 virulence factor nsp1 and the primosome, for which evidence first emerged from protein–protein interaction screens between SARS‐CoV‐2 and human proteins. Our structure shows that nsp1 uses its globular domain to interact with the primosome, distinct from the C‐terminal motif employed to target the ribosome and suppress mRNA translation. Thus, our findings provide the first structural evidence of how nsp1 can deploy its conserved middle domain to engage with a host protein.

The ability of nsp1 to use different regions of its sequence to target different physiological processes in the cell is in keeping with evidence of its pleiotropic pathological effects during infection. Furthermore, as viral proteins are known to exploit existing binding epitopes on the surface of the target protein, the nsp1‐binding site in Pol α might be indicative of as yet unknown physiological partners of the primosome binding at this site. The discovery of a novel protein–protein interaction surface in nsp1 and Pol α opens up the possibility of developing small‐molecule inhibitors aimed at disrupting their association, which might be of therapeutical value in the treatment of COVID‐19.

How the interaction of nsp1 with the primosome might promote virulence is currently unclear. However, a rationale for targeting the primosome by SARS‐CoV‐2 might be envisaged based on recent evidence that mutations in *POLA1*—coding for the catalytic subunit of Pol α—cause X‐linked reticulate pigmentary disorder (XLPDR), a genetic syndrome that is characterized by activation of type I IFN signaling, a persistent inflammatory state and recurrent lung infections.^32^, ^33^ A similar set of symptoms is observed in individuals with inherited haploinsufficiency of the *PRIM1* gene, coding for the Pri1 subunit of primase.^50^ In XLPDR fibroblasts, the ability of Pol α–primase to synthesize RNA/DNA hybrids in the cytoplasm was found to be impaired.^32^ This effect was proposed to be responsible for the disease, although the molecular mechanisms by which cytosolic RNA/DNA hybrids might help regulate the antiviral response are unknown.

To test whether nsp1 binding may directly affect the enzymatic activity of the primosome, we performed biochemical assays to measure the RNA and DNA primer synthesis activities of primase and Pol α, respectively. However, we could not detect an effect on RNA primer synthesis or DNA extension in the presence of an excess of nsp1 protein (Figure S8). This is consistent with the 3D structure of the primosome–nsp1 complex, in which nsp1 is docked in a position that is not expected to interfere with RNA–DNA primer synthesis.

**FIGURE 6:**
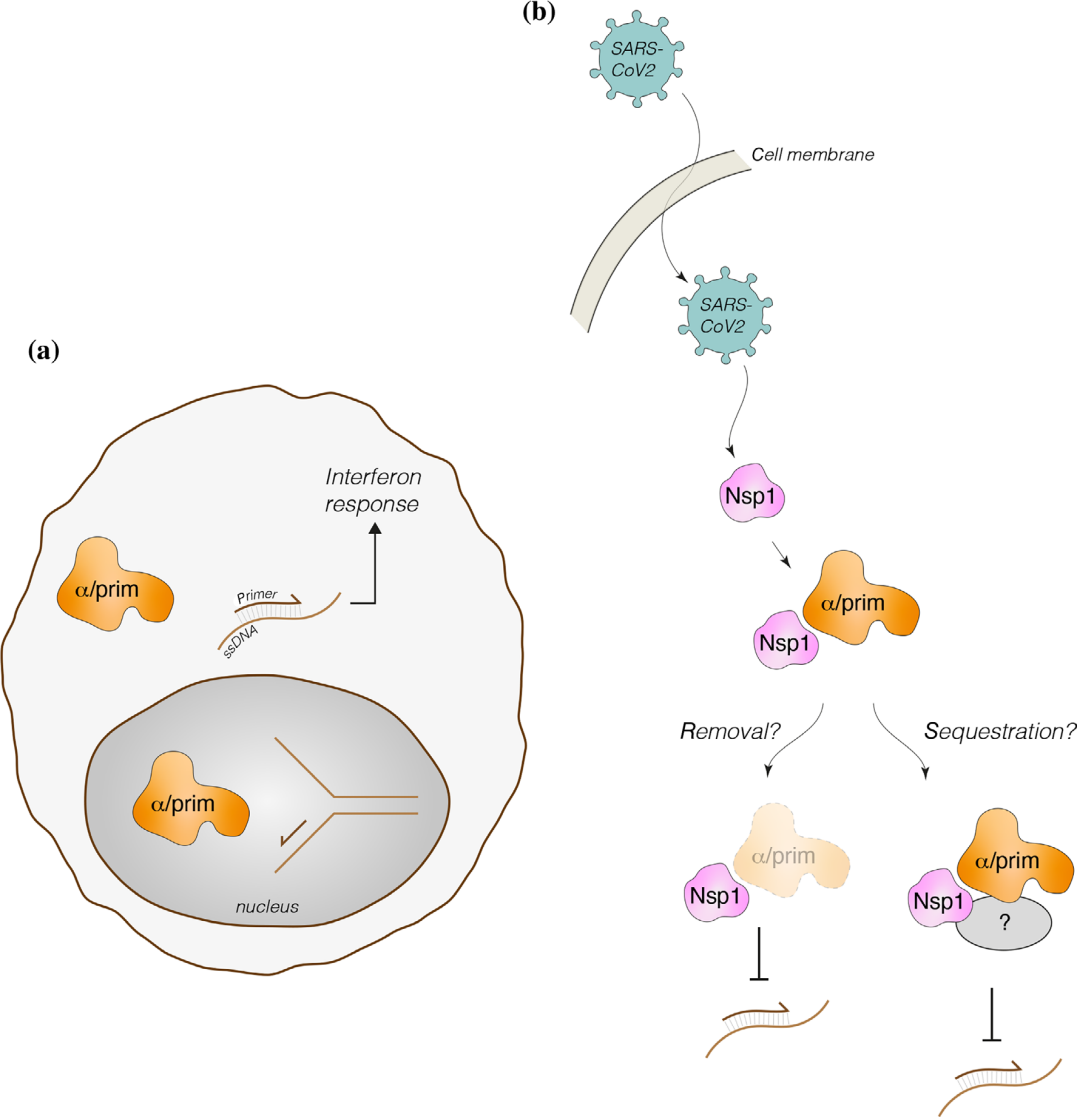
Possible functional significance of primosome targeting by SARS‐CoV‐2 nsp1. (a) In addition to its well‐established role in chromosomal DNA duplication during S‐phase, a cytoplasmic role for Pol α–primase has been postulated to synthesize RNA/DNA duplexes that might mediate the interferon response^32^; (b) speculative modes of action of nsp1‐dependent SARS‐CoV‐2 interference with cytoplasmic Pol α–primase. nsp1, Nonstructural protein 1; Pol α, DNA polymerase α

Given the exclusively cytoplasmic localization of SARS and MERS nsp1 upon infection,^30^ the interaction with nsp1 appears aimed at targeting the primosome fraction present in the cytoplasm of infected cells. As nsp1 binding did not seem to impact the enzymatic activity of the primosome, we speculate that any effect on primosome activity might be achieved indirectly. Thus, nsp1 binding might be necessary for recruitment of additional factors that inhibit primosome activity or sequester it in a way that makes it inactive; alternatively, nsp1 binding might target the primosome for ubiquitination and degradation (Figure 6). Further study will be required to establish how primosome targeting by SARS‐CoV‐2 affects the antiviral response of the host cell.

## 4 MATERIALS AND METHODS

### 4.1 Cloning, expression, and protein purification

Full‐length human primosome (Pol α: 1–1,462; B subunit: 1–598; PriS: 1–420; PriL: 1–509) and truncated human primosome (Pol α: 334–1,462; B subunit: 149–598; PriS: 1–420; PriL: 1–509) were expressed in *Sf9* insect cells and purified as described previously.^38^ Full‐length coronavirus nsp1 genes (SARS‐CoV‐2, SARS‐CoV, MERS, HKU1, NL63, and 229E) were ordered as synthetic gBlocks (IDT) and cloned into bacterial expression vector pMAT11.^51^ nsp1 proteins were expressed as His_6_‐MBP‐tagged fusion proteins from the Rosetta2 (DE3) *Escherichia coli* strain (Novagen). nsp1 fragments (SARS‐CoV N‐terminus [1–12], middle domain [13–127], and C‐terminus [128–180]; SARS‐CoV‐2 N‐terminus [1–12], middle domain [13–127], and C‐terminus [128–180]) were cloned and expressed in the same way. Full‐length SARS‐CoV‐2 nsp1 point mutants (V111D, L27D, and V28D) were ordered as synthetic gBlocks (IDT) and cloned and expressed as above.

All His_6_‐MBP‐fusion proteins were purified by metal affinity chromatography using Ni‐NTA agarose (Qiagen). A high‐salt wash (500 mM KCl) was performed on‐column to remove any traces of nucleic acid from the protein preparation. Proteins were eluted using 25 mM 4‐(2‐hydroxyethyl)‐1‐piperazineethanesulfonic acid (HEPES) pH 7.2, 300 mM KCl, and 200 mM imidazole.

### 4.2 Cryo‐EM sample preparation and grid freezing

SARS‐CoV His_6_‐MBP‐nsp1 (13–127) was TEV‐cleaved overnight at 4°C, and the His_6_‐MBP tag was subsequently recaptured using amylose resin (NEB). The flow‐through was loaded onto a Q‐HP anion exchange column (Cytiva) and eluted with a KCl gradient (25 mM HEPES pH 7.2, 80–750 mM KCl, and 2 mM Dithiothreitol [DTT]). Nsp1 and the truncated primosome were subsequently individually buffer‐exchanged into cryo‐EM buffer (25 mM HEPES pH 7.2, 150 mM KCl, and 1 mM DTT) using a NAP5 column (Cytiva). The two proteins were mixed at final concentrations of 1 μM primosome and 10 μM nsp1. 1 mM BS3 cross‐linker was added, and the sample was incubated on ice before grid freezing using a Vitrobot set at blot force −10 (Thermo Fisher Scientific). The samples were diluted 1:2 with cryo‐EM buffer immediately before the application of the protein sample to the grid; 3 μl sample was applied to each side of the UltrAuFoil grid (300 mesh, R 1.2/1.3, Quantifoil), which had been glow discharged on both sides (25 mA, 1 min each side). The grids used for the eventual structure determination were plunge frozen in liquid ethane approximately 45 min after the addition of the BS3 cross‐linker.

### 4.3 Cryo‐EM data processing and structure refinement

Data collection parameters, structure refinement details, and statistics are reported in Table S1 and Figure S1; 393 movies (1.11 e/Å^2^/fraction, 40 fractions) were collected on a Talos Arctica at 200 keV, 92,000× magnification, and 1.13 px/Å, with a Falcon III detector in counting mode. Movie processing was carried out in Relion 3.1.^52^ The movies were motion corrected using Relion's implementation and CTFs were estimated using CTFFIND4‐1.^53^ An initial set of 165,011 particles was auto‐picked and used to generate 2D templates for template‐based picking of a new set of 183,301 particles. The template‐picked particle set was reduced to 97,680 particles by rounds of 2D classification and used to reconstruct an initial 3D volume. 3D classification into four models generated one model with 47,542 particles (48.6%) which was refined to 7.29 Å. The 3D volume showed clear evidence of the four primosome subunits and of the bound nsp1 protein; the initial 3D model was used as starting point for 3D classification during processing of the higher resolution data set.

A total of 2,919 movies (0.98 e/Å^2^/fraction, 48 fractions) were collected on Titan Krios at 300 keV, 130,000 magnification, and 0.326 Å px/Å with a K3 detector in super‐resolution counting mode. Movie processing was carried out in Relion 3.1.^52^ The movies were motion corrected and 2× binned using Relion's own implementation, and CTFs were estimated using CTFFIND4‐1.^53^ An initial set of 314,987 particles was auto‐picked and improved by rounds of 2D classification, to generate 2D templates for template‐based picking of a new set of 709,068 particles. The particle set was improved by multiple rounds of selection by 2D classification, and a first 3D classification into four models was performed using the initial 3D model obtained for the Talos data set. Class #1 (251,901 particles) was selected and a second round of 3D classification was performed, leading to a new 3D Class #4 with 233,476 particles. 3D refinement of this class yielded a map at 4.12 Å, which was sharpened in Phenix^54^ using “phenix.local_aniso_sharpen” to a resolution of 3.8 Å (0.143 FSC) and used for model interpretation and model building.

For model building, the crystal structure of human Pol α–primase (PDB ID 5EXR) was fitted manually in the map with ChimeraX^55^ and real‐space refined in Phenix,^54^ together with local manual rebuilding in Coot.^56^ Additional density corresponding to the known structure of nsp1 was clearly detectable, although at a lower map threshold, bound to Pol α. A model of SARS nsp1 was built based on PDB entries 7K3N and 2HSX and docked into the map in ChimeraX.^55^ The Pol α–primase–nsp1 complex was further real‐space refined in Phenix.^54^

Inspection of the 2D/3D classes and of the 3D refined map for the Pol α–primase–nsp1 complex showed that the density corresponding to the Pri1 subunit of primase was weaker. This observation was explained by the looser contact of Pri1 with the rest of the Pol α–primase, which is primarily mediated by the known interface with Pri2.^42^ To improve the map of the rest of Pol α–primase and of the bound nsp1, a mask was prepared in ChimeraX^55^ that excluded the Pri1 lobe and used to generate a set of subtracted particles from the 233,476 particles set used to generate the original 3D refined map. A new initial 3D model was first generated, and after a round of 3D classification, Class #3 (103,763 particles) was selected. 3D refinement of this class generated a 4.40 Å map, that was sharpened using “phenix.local_aniso_sharpen” to a resolution of 3.6 Å (01.43 FSC) and used for model interpretation and model building. The map showed slightly better high‐resolution features for the primosome core and clearer features for the nsp1 fold and was therefore used as a visual aid in the refinement of the primosome–nsp1 complex structure.

### 4.4 Pull‐downs

A total of 150 μl amylose resin (NEB) was equilibrated in pull‐down buffer (1× phosphate‐buffered saline, 5% glycerol, 0.1% Igepal CA‐630, and 1 mM tris(2‐carboxyethyl)phosphine or DTT). An excess of His_6_‐MBP‐nsp1 was added to saturate the beads with bait protein. The His_6_‐MBP tag protein was used as negative control. Beads were washed twice with pull‐down buffer to remove excess, unbound bait protein. BSA was added to a final concentration of 2.5 mg/ml^−1^. Primosome (full length or truncated) was added to the beads, which were subsequently incubated at 4°C for 1 hr with rolling. The beads were washed four times with 1 ml pull‐down buffer. Bound protein was eluted using 150 μl pull‐down buffer supplemented with 50 mM maltose. Samples were analyzed by sodium dodecyl sulfate–polyacrylamide gel electrophoresis.

### 4.5 Primosome activity assay

Full‐length His_6_‐MBP‐nsp1 (from SARS‐CoV‐2 and SARS‐CoV) was TEV‐cleaved overnight at 4°C. The His_6_‐MBP tag was recaptured using amylose resin (NEB). The flow‐through was loaded onto a Q‐HP anion exchange column (Cytiva), and nsp1 was eluted with a KCl gradient (25 mM HEPES pH 7.2, 80–750 mM KCl, and 2 mM DTT). Pure nsp1 was subsequently buffer exchanged into 25 mM HEPES pH 7.2, 100 mM KCl, and 1 mM DTT using a NAP5 column (Cytiva).

An activity assay, to monitor the ability of Pol α to extend an RNA primer, was performed using a pre‐annealed DNA–RNA template (DNA45: 5′‐ TTCTTT ATCTCTTATTTCCTTCTATTTTCCACGCGCCTTCTATTT‐3′; RNA13: 5′‐[6FAM]‐GGCGCGUGGAAAA‐3′, Sigma Aldrich). The reaction buffer comprised 25 mM HEPES pH 7.2, 100 mM KCl, 10 mM Mg(OAc)_2_, and 1 mM DTT. Each 100 μl reaction contained 2 μM DNA–RNA template, 1 mM dNTP, 10 μM nsp1, and 0.1 μM full‐length primosome. Reactions were incubated at 25°C, and 30‐μl aliquots were removed and quenched (by adding an equal volume of 95% formamide and 25 mM EDTA) at the indicated time points. Samples were heated to 70°C for 2 min, then loaded onto a 19% urea‐polyacrylamide gel (running conditions: 450 V, 90 min, 0.5× TBE buffer). Gels were initially imaged by laser scanning (473 nm laser, Typhoon FLA 9000, GE Healthcare), to detect only the 6FAM‐labeled products. Gels were subsequently stained using a 1:10000 dilution of SYBR Gold Stain (Thermo Fisher Scientific) in 0.5× TBE buffer and reimaged in the same way to detect all nucleic acid.

## CONFLICT OF INTERESTS

The authors declare no conflict of interest.

## AUTHOR CONTRIBUTIONS

**Mairi L Kilkenny:** Conceptualization (equal); data curation (lead); investigation (lead); supervision (equal); writing – original draft (lead); writing – review and editing (equal). **Charlotte E Veale:** Data curation (supporting); investigation (supporting). **Amir Guppy:** Data curation (supporting); investigation (supporting). **Steven W Hardwick:** Data curation (supporting). **Dimitri Y Chirgadze:** Data curation (supporting). **Neil J Rzechorzek:** Data curation (supporting); investigation (supporting); writing – review and editing (supporting). **Joseph D Maman:** Data curation (supporting); investigation (supporting); writing – review and editing (supporting). **Luca Pellegrini:** Conceptualization (equal); funding acquisition (lead); project administration (lead); supervision (lead); writing – original draft (equal); writing – review and editing (lead).

## Supporting information


**Appendix** S1: Supporting Information

## Data Availability

Coordinates and 3D reconstructions for the primosome–nsp1 complex have been deposited under accession codes 7OPL (PDB), 13020 (EMDB), and 13021 (EMDB).
